# A Novel ZNF304/miR-183-5p/FOXO4 Pathway Regulates Cell Proliferation in Clear Cell Renal Carcinoma

**DOI:** 10.3389/fonc.2021.710525

**Published:** 2021-10-07

**Authors:** Li-Xin Ren, Bo-Wen Zeng, Meng Zhu, An-Ning Zhao, Bei Shi, Hong Zhang, Dan-Dan Wang, Jun-Fei Gu, Zhan Yang

**Affiliations:** ^1^ Department of Urology, The Second Hospital of Hebei Medical University, Shijiazhuang, China; ^2^ Department of Urology, Affiliated Hospital of Sergeant School of Army Medical University, Shijiazhuang, China

**Keywords:** zinc-finger protein 304, proliferation, FOXO4, miR-183-5p, clear cell renal carcinoma (ccRCC)

## Abstract

Zinc-finger protein 304 (ZNF304) plays a critical role in silencing genes through transcription, regulating cell survival, proliferation, apoptosis, and differentiation during development. However, the roles of transcription factor ZNF304 and its clinical significance in clear cell renal carcinoma (ccRCC) remain unclear. In this study, we found that the expression of ZNF304 was downregulated in ccRCC tissues. Lower levels of ZNF304 were correlated with poor survival. Downregulation of ZNF304 promoted ccRCC cell growth *in vitro*, whereas overexpression of ZNF304 inhibited growth. Our results indicated that miR-183-5p/FOXO4 mediated ZNF304 regulation of cell growth. Interestingly, we revealed that ZNF304 promoted FOXO4 expression in ccRCC cells. Mechanistically, ZNF304 binds to miR-183 promoter and inhibits miR-183-5p transcription. Furthermore, the expression of miR-183-5p wes increased in ccRCC tissues, and the upregulation of miR-183-5p was related to the poor prognosis of ccRCC patients. miR-183-5p upregulation repressed the expression of FOXO4 and promoted ccRCC progression. These results demonstrated that ZNF304/miR-183-5p/FOXO4 axis played essential role in promoting ccRCC progression, which suggests that disruption of this axis may be a potential therapeutic target in ccRCC.

## Introduction

Renal cell carcinoma (RCC), which is one of the most common malignant tumors in humans, is also the most deadly urinary tract tumor (1). In 2020, there were 76,080 new RCC cases and more than 13,000 deaths in the United States (2). Moreover, the incidence and mortality caused by RCC are increasing. Among them, clear cell RCC (ccRCC) accounts for 75%–80% of renal cell carcinoma cases, and is the most common histological subtype in renal cell carcinoma (3, 4). Surgical resection of the tumor usually prolongs progression-free and overall survival of patients (5, 6). However, more than 25% of patients have developed the metastatic RCC (mRCC) at the time of diagnosis (7). Unfortunately, the clinical results for kidney cancer patients were not satisfactory in the past decade; even though tyrosine kinase inhibitor (CTKI) drugs, rapamycin protein (mTOR) inhibitor drugs and immunotherapy showed promise for treatment (8–10), 30% of newly diagnosed locally advanced kidney cancer patients will metastasize (11). Therefore, understanding the molecular mechanisms underlying malignant ccRCC is of great significance, as it will aide in determining new drug targets and treatment strategies.

The occurrence and development of tumors is a complex process involving the abnormal expression of multiple genes, such as up-regulated proto-oncogenes and down-regulated tumor suppressor factors (12–15). Unfortunately, many current studies have focused on up-regulated genes in tumors, and have ignored the research on down-regulated genes. In fact, these abnormal genes play an equally important role in the tumor process (16). Zinc-finger protein 304 (ZNF304), which is a transcription factor, belongs to the Krueppel C2H2-type zinc-finger family protein (17). ZNF304 often function as f transcriptional repressor and plays a role in gene silencing (18). ZNF304 binds to the promoters of INK4-ARF and other CpG island methylator phenotype genes, and facilitates transcriptional silencing by recruiting co-repressor complexes (19). ZNF304 binds to the ITGB1 promoter and upregulates the transcription of β-1 integrin (18). However, the expression and function of ZNF304 and its downstream effectors in ccRCC are not well understood.

Forkhead box O 4 (FOXO4) transcription factor is one of the Forkhead box O family proteins which also contain FOXO1, FOXO3a, and FOXO6 (20). FOXO factors are involved in the regulation of the insulin signaling pathway, and implicated in regulating cellular processes such as cell viability, proliferation, energy production and immune responses (21). FOXO4 acts as a tumor suppressor in multiple human cancers, such as lung, leukemia, cervical, pancreatic and CRCs etc. (22–24). In glioblastomas, FOXO4 inhibits the malignant phenotype of cells *in vitro* and *in vivo* (25). Importantly, overexpression of FOXO4 promotes apoptosis of ccRCC cells by repressing Bim expression (26).

Here, we demonstrate that ZNF304 and FOXO4 are downregulated in ccRCC tissue, where lower levels of these proteins are associated with poor prognosis of patient survival. Downregulation of ZNF304 promotes ccRCC cell growth *in vitro*. Mechanistically, ZNF304 bound directly to the miR-183 promoter and inhibited miR-183-5p expression by transcriptional activation. The results revealed that ZNF304/miR-183-5p/FOXO4 axis plays a critical key role in ccRCC growth and may serve as a potential therapeutic target.

## Methods

### Clinical Samples

Human primary clear cell renal cell carcinoma and corresponding normal kidney tissue were collected from the Department of Urology, the Second Hospital of Hebei Medical University. All patients were ccRCC patients from July 2015 to June 2020. All patients underwent radical nephrectomy for treatment. Fresh ccRCC and corresponding normal kidney tissue were washed with saline and quickly frozen in liquid nitrogen or formaldehyde to fix for further used. The research protocol has been approved by the Ethics Committee of the Second Hospital of Hebei Medical University, and each patient’s written consent has been obtained.

### Cell Lines and Transfection

Human ccRCC cell lines (SW839, A498, Caki-1, 786-0) were purchased from ATCC (Rockville, Maryland, USA), and are preserved in our laboratory. The 293A cell line is deposited in our laboratory. The above-mentioned cells were cultured in low-sugar dulbecco’s modified eagle medium (DMEM) supplemented with 10% fetal bovine serum (Clark Bio, Claymont, DE, USA) and 1% penicillin/streptomycin (Solarbio, Beijing). The cells are cultured in a humidified condition of 95% air and 5% CO_2_. All cells were transfected using Lipofectamine 2000 (Invitrogen) according to the manufacturer’s operating manual. Briefly (27), 786-O and SW839 cells (1×10^5^ cells/ml) were seeded into plates in growth medium (DMEM). Until the required number of cells (80% confluence) is obtained at the time of transfection. The cells were washed twice with PBS and the transfected lipoplex (prepared as described in the operating manual) was added to each well. It was mixed gently by rocking the plate back and forth. The cells were cultured in DMEM (without FBS), 5% CO_2_, and 37°C humidified incubator for 4-6 hours. Then the cells were washed twice with PBS, replaced with complete medium, and cultured for 48 hours.

### RNA Isolation and RT-qPCR

Use RNAeasy Mini Elute Kit (QIAGEN) according to the manufacturer’s manual for total RNA isolation. The NanoDrop 2000 system detects RNA concentration and quality. The first strand of cDNA was synthesized using M-MLV First Strand Kit (Life Technologies) with random hexamer primers. Dilute the first strand of cDNA 5-10 times as needed, and use Platinum SYBR Green qPCR Super Mix UDG kit (Invitrogen) to perform real-time quantitative PCR (qRT-PCR) analysis on mRNA in ABI 7500 FAST System (Life Technologies). The relative transcriptional expression level of gene mRNA was standardized with GAPDH as an internal reference gene. The calculation was carried out using the 2^-ΔΔCt^ formula according to the previous description (28). ZNF304-F:GACCGGGTTCAGAGTTGTGT; ZNF304-R:CCACGTGTGCACAGTTTCTG; FOXO4-F:GGGAAAAGGCCATTGAAAGCG; FOXO4-R:TGTGGCGGATCGAGTTCTTC; GAPDH-F:ATGAATGGGCAGCCGTTAGG; GAPDH-R:TGGAATTTGCCATGGGTGGA; Cyclin D1-F:CTGATTGGACAGGCATGGGT; Cyclin D1-R:GTGCCTGGAAGTCAACGGTA; miR-23-3p-F:GGCACATTGCCAGGGATTTC; miR-96-5p-F:GGTTTGGCACTAGCACATTTTTG; miR-128-3p-F:GGCTCACAGTGAACCGGTCTC; miR-142-3p-F:GGCCGTAGTGTTTCCTACTTTATG; miR-150-5p-F:GCTCTCCCAACCCTTGTACCAG; miR-183-5p-F:GGCTATGGCACTGGTAGAATTCAC; miR-216b-5p-F:GCAAATCTCTGCAGGCAAATGTG; miR-217-F:GTACTGCATCAGGAACTGATTGG; miR-449a-5p-F:GGTGGCAGTGTATTGTTAGCTGG; miR-1271-5p-F:CTTGGCACCTAGCAAGCACTC.

### Western Blot Analysis

Western blot analysis was performed as previously described (28). Polyvinylidene fluoride (PVDF) membranes (Millipore), were incubated with primary antibodies anti-ZNF304 (1:500, HPA050531), anti-FOXO4 (1:1000, ab128908), anti-cyclin D1 (1:500, 26939-1-AP), and anti-β-actin (1:1000, sc-47778). An HRP-conjugated secondary antibody (1:5000, Rockland) was used and detection was by ECL (enhanced chemiluminescence) Fuazon Fx (Vilber Lourmat). Images were captured and processed by FusionCapt Advance Fx5 software (Vilber Lourmat). All experiments were repeated three times.

### Vector Construction and Luciferase Reporter Assay

Lentiviral pLKO-ZNF304 (shZNF304), pWPI-ZNF304 (overexpression ZNF304, oeZNF304) and pLKO-FOXO4 (shFOXO4) plasmids were designed and constructed from Biocaring Biotechnology Co., Ltd (Shijiazhuang). Briefly (28), ZNF304 cDNA was inserted into EcoRI and XhoI restriction endonuclease digested pWPI empty vector (Addgene, #12254) and then Sanger sequencing for confirming. ZNF304 and FOXO4 shRNA were inserted into BsmBI restriction endonuclease digested pLKO (Addgene, #14748) and then Sanger sequencing for confirming. The 3’ UTR sequences of FOXO4 containing wild-type (WT) or mutant (mut) forms of the miR-183-5p target site were inserted into restriction endonucleases XhoI (Xho1) and SalI (Sal1) along with pmirGLO Dual-Luciferase miRNA Target Expression Vector (Promega, Madison, WI, USA). FOXO4 reporter construct (WT or mut) or the empty reporter vector were co-transfected into SW839 cells with miR-183-5p mimic or mimic control and plasmid Renilla luciferase-thymidine kinase. Luciferase activity was measured by Dual-Glo Luciferase Assay System (Promega) with a Flash and Glow reader (LB955; Berthold Technologies, Bad Wildbad, Germany). The specific target activity was expressed as the relative activity ratio of firefly luciferase to Renilla luciferase.

### Morphometry and Histology

Human ccRCC and normal kidney tissues were fixed in formalin and then processed for routine paraffin embedding (29). Ten 5-μm-thick consecutive sections were prepared for hematoxylin and eosin staining. Images were acquired using a Leica microscope (Leica DM6000B, Switzerland) and digitized with LAS V.4.4 (Leica).

### MTT Assay

Cell viability was detected by an MTT [3-(4,5-dimethylthiazol-2-yl)-2,5-diphenyltetrazolium bromide] colorimetric assay as previously described (29). Briefly, SW839 and 786-0 cells were plated and transfected with the indicated vector or treated with AZD6244 for 24 h. Absorbance at 570nm was measured using a microplate reader (Thermo Fisher, USA).

### Colony Formation Assay

100 cells/well were seeded into six-well plates, cultured for 1 week and fixed in a glacial acetic acid/methanol solution. Then, 0.5% crystal violet was used to stain the colonies. Colony numbers were counted under a microscope (30).

### ChIP Assay

The chromatin immunoprecipitation (ChIP) assay was performed as previously described (30). Briefly, SW839 cells were treated with formaldehyde. The cross-linked chromatin was sonicated to an average size of 400–600 bp. Samples were precleared with protein A-agarose/salmon sperm DNA and the DNA fragments were immunoprecipitated overnight at 4°C with anti-ZNF304 or anti-IgG antibodies. After reversal of cross-linking, ZNF304 occupancy on the miR-183-5p promoter was examined. Results were determined by qRT-PCR with miR-183-prom-F1: GGGCCTTCAGGTGGAGATAGGAG; miR-183-prom-R1:CCCTGCGGAGAGACCAGCGG; miR-183-prom-F2:CAGTCTGGCCCAATCTGGTCTGG; miR-183-prom-R2:GGAGAGGCAAGCAGCTGGCCAG; miR-183-prom-F3:GGGTGCCAGCTCCCCAGAGACC; miR-183-prom-R3:CTGCAGCCACCCAGGTGGCTTG.

### Statistical Analysis

Statistical analysis as previously described (28). Data were presented as the mean ± SEM. Student’s t test was used to analyze differences between two groups. Spearman’s correlation analysis was used to evaluate the correlation analysis. Values of p < 0.05 were considered statistically significant. GraphPad Prism 7.0 software was used for the statistical analysis (GraphPad Software).

## Results

### The Expression of ZNF304 Is Down-Regulated in ccRCC Tissue, and the Lower Level of ZNF304 Indicates a Poor Prognosis for Patients

**Figure 1:**
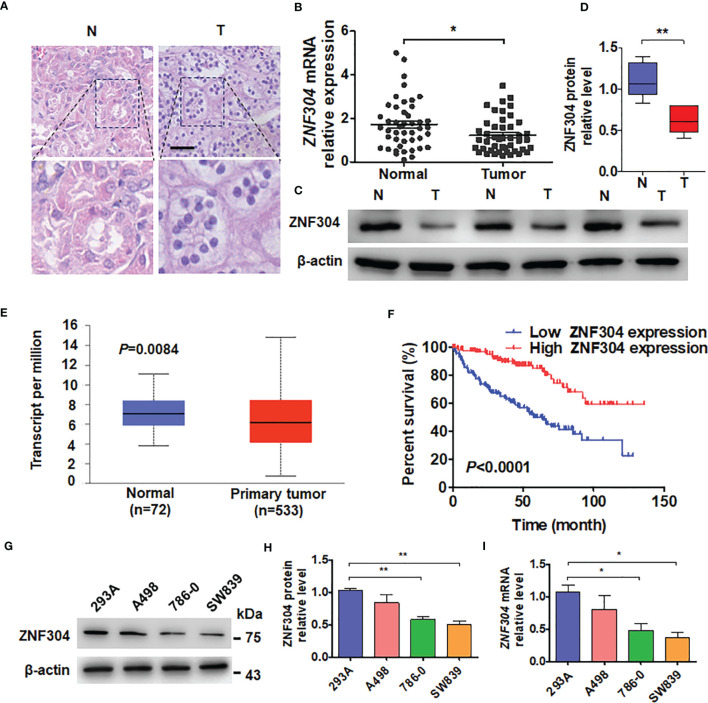
ZNF304 downregulation in ccRCC is correlated with good prognosis. **(A)** Staining of Hematoxylin and eosin in ccRCC (T) and normal (N) kidney tissues. Scale Bar = 50 μm. **(B)** RT-qPCR was use to examine ZNF304 mRNA level in ccRCC (T, n = 43) and normal kidney (N, n = 43) tissues. **(C)** Western blot analysis detected the protein levels of ZNF304 in ccRCC (T) and normal (N) kidney tissues **(D)** Quantitative analysis of **(C)**. **(E)** ZNF304 mRNA levels were analyzed from data of the TCGA database. **(F)** Kaplan–Meier analysis of the overall survival of ccRCC patients in the TCGA database with low (n = 130) and high (n = 130) ZNF304 levels (cutoff value is 25%). **(G)** Western blot was used to measure the proteins level of ZNF304 in 293A and RCC cell lines (A498, 786-0, and SW839). **(H)** Quantitative analysis of **(G)**. **(I)** qRT-PCR was performed to detect the ZNF304 mRNA level in the above cell lines. All data are from three independent experiments and expressed as the mean ± SEM. *P < 0.05, **P < 0.01 vs. the corresponding controls.

To investigate the expression level of ZNF304 in ccRCC, firstly, we collected clinical samples and located the ccRCC tumor and an area of normal kidney tissue by using hematoxylin and eosin staining (**Figure 1A**). Compared with normal kidney tissue, the mRNA and protein levels of ZNF304 in ccRCC tissue (T) are significantly reduced (**Figures 1B–D**). Data from the TCGA database show that the mRNA expression level of ZNF304 in ccRCC tissue is significantly lower than that in normal kidney tissue (**Figure 1E**). And the higher levels of ZNF304 indicates a good prognosis for patients in patients with ccRCC (**Figure 1F**). Furthermore, clinicopathologic factors of ZNF304 mRNA expression level was markedly negative associated with tumor grade but not with age, sex and tumor size etc (**Table 1**). Next, we detected ZNF304 expression in cell lines. 786-0 and SW839 cells expressed much lower levels of ZNF304 compared with the other cell lines (**Figures 1G–I**). These results suggest that downregulation of ZNF304 may be associated with ccRCC progression.

**Table 1:** Clinicopathological characteristics.

Characteristics	Number of patients (%)	ZNF304 expression		
		Low (%)	High (%)	*P* value
**No. of patients**	45	22	23	
**Age**				
≤60	22	11 (50.00)	11 (50.00)	1.000
>60	23	11 (47.83)	12 (52.17)	
**Gender**				
Male	30	16 (53.33)	14 (46.67)	0.530
Female	15	6 (40.00)	9 (60.00)	
**Tumor size (**cm**)**				
≤5	27	12 (44.44)	15 (55.56)	0.550
>5	18	10 (55.56)	8 (44.44)	
**pT status**				
pT_1_–pT_2_	18	11 (61.11)	7 (38.89)	0.125
pT_3_–pT_4_	27	9 (33.33)	18 (66.67)	
**pN status**				
pN0	30	14 (46.67)	16 (53.33)	0.758
pN1–pN3	15	8 (53.33)	7 (46.67)	
**TNM stage**				
I–II	28	10 (35.71)	18 (64.29)	0.033
III–IV	17	12 (70.59)	5 (29.41)	

### ZNF304 Inhibits Cell Proliferation in ccRCC

**Figure 2:**
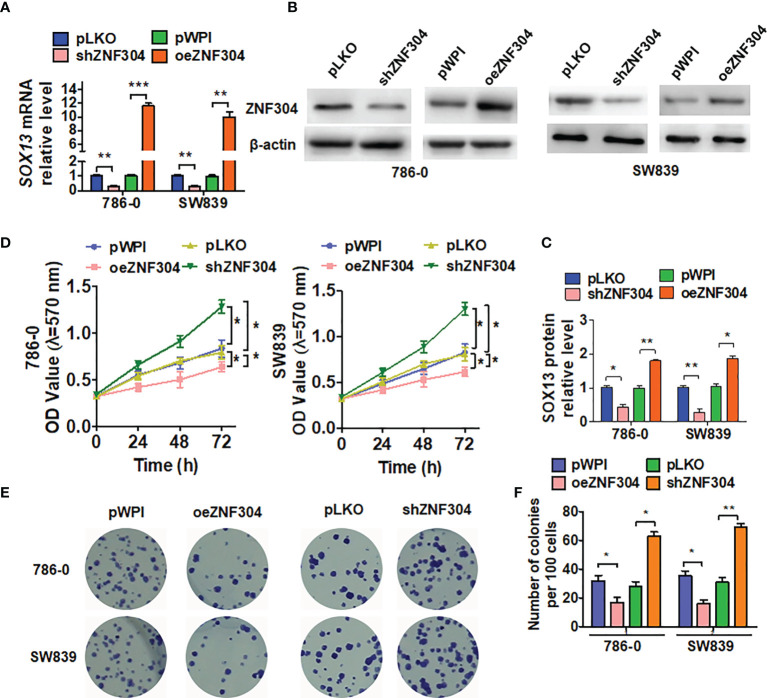
ZNF304 inhibits ccRCC cell growth. **(A)** The shZNF304, oeZNF304 and control vectors were transfected to SW839 and 786-0 cell, and then qRT-PCR was used to examine the mRNA level of ZNF304. **(B)** SW839 and 786-0 cells were transfected as in **(A)**, and the protein level ZNF304 was measured by Western blot. **(C)** Quantitative analysis of **(B)**. **(D–F)** Cells were transfected as in **(A)**, and cell viability was examined by MTT **(D)** and colony formation assays **(E, F)**. All data are from three independent experiments and expressed as the mean ± SEM. *P < 0.05, **P < 0.01, ***P < 0.001 vs. the corresponding controls.

Previous studies revealed ZNF304 to be a transcriptional repressor involved in cancer cell survival (18). Overexpression of ZNF304 with a lentiviral vector markedly increased ZNF304 mRNA and protein levels, whereas transfection with shZNF304 significantly reduced ZNF304 expression (**Figures 2A–C**). The MTT assay showed that ZNF304 overexpression inhibited growth of both 786-0 and SW839 cells, whereas ZNF304 downregulation accelerated cell line proliferation (**Figure 2D**). The colony formation assay confirmed these findings (**Figures 2E, F**). Taken together, these data reveal that ZNF304 inhibits ccRCC cell growth.

### ZNF304 Repression of Cell Growth *In Vitro* Is Mediated by FOXO4

**Figure 3:**
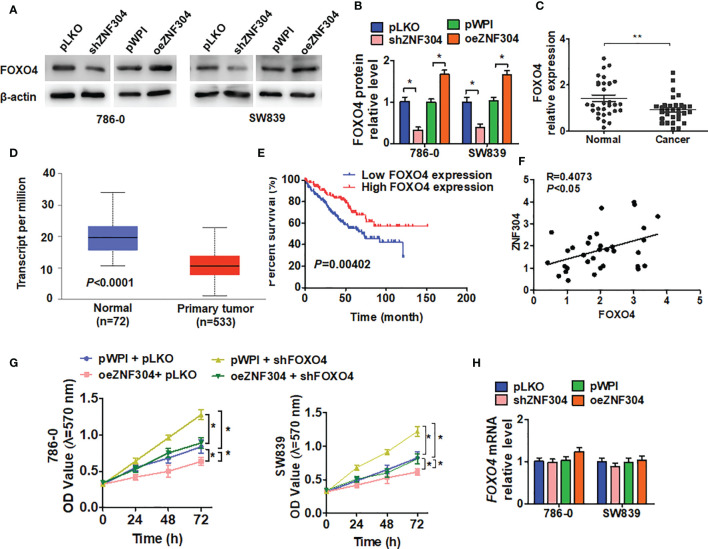
FOXO4 regulates ZNF304-mediated cell proliferation. **(A)** 786-0 and SW839 cells were transfected with pKLO, shZNF304, pWPI, and oeZNF304 vectors, and then protein expression of FOXO4 was measured by western blot. **(B)** Quantitative analysis of **(A)**. **(C)** The expression of FOXO4 mRNA was measured by qRT-PCR in ccRCC (T, n = 43) and normal kidney (N, n = 43) tissues. **(D)** the overall survival of ccRCC patients from the TCGA database was analyzed by Kaplan–Meier analysis. FOXO4 levels low (n = 130) and high (n = 130) (cutoff value is 25%). **(E)** The correlation between ZNF304 and FOXO4 mRNA expression in PCa tissues was analyzed by Pearson correlation analysis of our clinical data (R = 0.4073, *P* < 0.05). **(F)** 786-0 and SW839 cells were transfected with oeZNF304, shFOXO4, oeZNF304 + shFOXO4 and control vectors, and cell viability was measured by MTT. **(G)** Cells were prepared as in **(A)**, and FOXO4 mRNA was detected by RT-qPCR. **(H)** Cells were prepared as in **(A)**, and cell viability was measured by MTT **(D)** and colony formation assays **(E, F)**. All data are from three independent experiments and expressed as the mean ± SEM. *P < 0.05, **P < 0.01 vs. the corresponding controls.

To elucidate the molecular pathway underlying ccRCC cell growth regulation by ZNF304, we analyzed the correlation of gene expression with ZNF304 in ccRCC tissues from the TCGA database (**Supplementary Figure 1**). The transcription factor FOXO4 appeared to be implicated in this. Knockdown of ZNF304 reduced FOXO4 protein level while ZNF304 overexpression upregulated FOXO4 (**Figures 3A, B**). FOXO4 was also detected in ccRCC and normal kidney tissues. Compared to normal kidney tissues, the expression of FOXO4 was obviously decreased in ccRCC tissues (T) (**Figure 3C**). The data from TCGA databases also show that FOXO4 mRNA levels were downregulated in ccRCC (**Figure 3D**). And the lower expression level of FOXO4 was associated with poor overall survival in ccRCC patients (**Figure 3E**). Moreover, we confirmed that FOXO4 has a positive correlation with ZNF304 in ccRCC using PCR data (**Figure 3F**). The MTT assay showed that depletion of FOXO4 facilitated cell proliferation and reversed the inhibition caused by ZNF304 overexpression (**Figure 3G**). Surprisingly, overexpression or depletion of ZNF304 did not affect FOXO4 mRNA levels (**Figure 3H**). These findings show that FOXO4 underlies the regulation of ccRCC cell proliferation by ZNF304.

### miR-183-5p Mediates ZNF304-Promoted FOXO4 Expression

**Figure 4:**
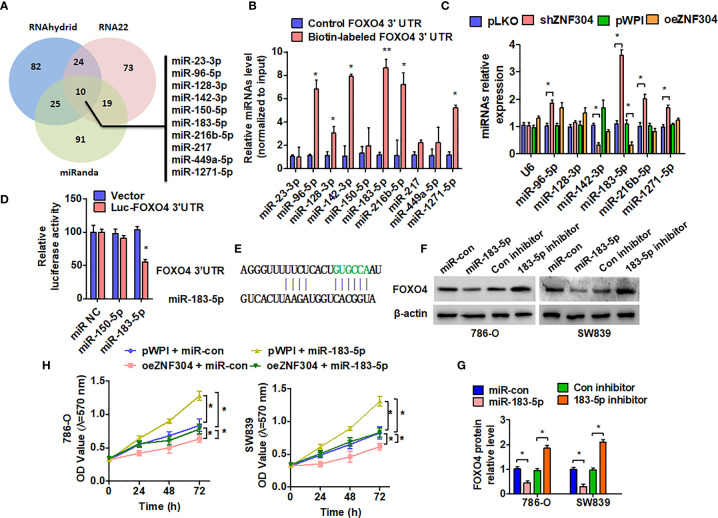
miR-183-5p mediates ZNF304-regulated FOXO4 expression in ccRCC. **(A)** Potential microRNAs targeted 3′UTR of FOXO4 were predicted in three online target-prediction programs, and showed with venn diagram. **(B)** Biotin pull-down assay was used to enrich miRNAs, and RT-qPCR was used to detect these candidate miRNAs. **(C)** 786-0 cells were transfected with oeZNF304 or shZNF304 or control vectors. Candidate miRNAs were detected by using RT-qPCR. U6 served as a negative control. **(D)** 786-0 cells were co-transfected with the FOXO4 3′UTR and miR-183-5p or miR-150-5p mimics. Luciferase reporter assays showed that miR-183-5p reduced FOXO4 3′UTR luciferase activity. **(E)** The prediction of miR-183-5p binding site in the FOXO4 3′UTR. **(F, G)** 786-0 and SW839 cells were transfected with the indicated miRNAs, and then FOXO4 protein expression was measured by western blotting. **(H)** 786-0 and SW839 cells were co-transfected with the indicated miRNAs and vectors, and cell viability was measured by MTT. All data are from three independent experiments and expressed as the mean ± SEM. *P < 0.05, **P < 0.01 vs. the corresponding controls.

Regulation of FOXO4 protein expression level by ZNF304, without influence in FOXO4 mRNA, suggests that ZNF304 regulates FOXO4 at the epigenetic regulation. Because microRNAs (miRNAs) are one of critical regulators in the regulation of gene expression at the posttranscriptional level, we predicted miRNAs that may target the FOXO4 3′-UTR from TargetScan, miRanda, and RNA22 database (**Figure 4A**). FOXO4 3′-UTR containing biotin-labeled uracil was generated by a T7 RNA transcriptase. Then, we performed a pull-down assay combined with RT-PCR to detect the enrichment of these candidate miRNA levels (**Figure 4B**). The result showed that 6 high enrichment miRNAs were changed after up- and downregulation of ZNF304. However, only miR-183-5p was increased in shZNF304 transfected cells and decreased in ZNF304 overexpressing cells (**Figure 4C**). In addition, the luciferase reporter assay demonstrated that miR-183-5p can target the 3′UTR of FOXO4 (**Figures 4D, E**). Furthermore, western blot also confirmed that FOXO4 was a directly target of miR-183-5p (**Figures 4F, G**). miR-183-5p mimic facilitated cell proliferation and reversed cell growth inhibition caused by ZNF304 overexpression (**Figure 4H**). Totally, these finding indicated that miR-183-5p, which was regulated by ZNF304, directly targets 3′URT and repressed FOXO4 expression.

### ZNF304 Regulates miR-183-5p Transcription

**Figure 5:**
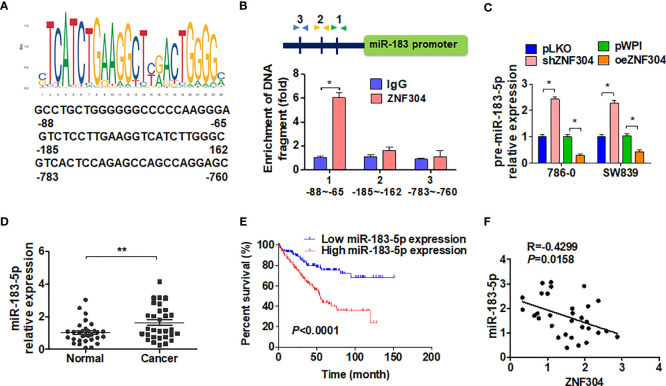
ZNF304 downregulates miR-183-5p expression by transcriptional repression. **(A)** The level of miR-183-5p was detected by qRT-PCR in ccRCC (T, n = 43) and normal kidney (N, n = 43) tissues. **(B)** According to the expression level of miR-183-5p in the TCGA database, kaplan-Meier was used to analyze the overall survival of ccRCC patients (cutoff value is 25%). **(C)** Pearson correlation analysis ZNF304 and miR-183-5p correlation (R = 0.4299, *P* = 0.0158). **(D)** JASPAR (http://jaspar.genereg.net/) predicted the potential binding site for ZNF304 in the miR-183-5p promotor. **(E)** Binding site of ZNF304 in miR-183-5p promoter region was confirmed by using ChIP-qPCR in 786-0 cell. The position of the primer is indicated by the arrowheads. **(F)** 786-0 and SW839 cells were transfected with oeZNF304 or shZNF304 or control vectors, and pre-miR-183-5p expression was measured by RT-qPCR. All data are from three independent experiments and expressed as the mean ± SEM. *P < 0.05, **P < 0.01 vs. the corresponding controls.

In order to confirm whether ZNF304 directly regulated miR-183-5p expression through transcription, we first analyzed the potential ZNF304 binding sites in the miR-183 2-kb 5’-promoter region on the nsembl and PROMO 3.0 websites. The results showed that there were three potential binding sites for ZNF304 in this region. (**Figure 5A**). ChIP analysis proved that ZNF304 mainly bound to the -80 to -65 bp region of the transcription start site in the miR-183 promoter (**Figure 5B**). Furthermore, in RCC cells where ZNF304 was downregulated pre-miR-183-5p expression was promoted and ZNF304 downregulation inhibited pre-miR-183-5p expression (**Figure 5C**). Compare to the normal kidney tissues, miR-183-5p level was significantly upregulation in ccRCC tissues (T) (**Figure 5D**). And the higher expression level of miR-183-5p in ccRCC patients were associated with poor overall survival (**Figure 5E**). Additionally, the mRNA level of miR-183-5p was positively correlated with ZNF304 in ccRCC tissues (**Figure 5F**). These findings reveal that ZNF304 directly promotes miR-183-5p transcription.

### Disruption of ZNF304/miR-183-5p/FOXO4 Axis Inhibits ccRCC Progression

**Figure 6:**
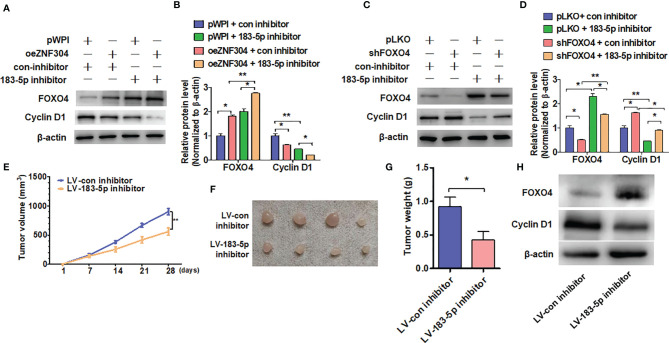
ZNF304/miR-183-5p/FOXO4 axis inhibits ccRCC progression. **(A, B)** 786-0 cells were transfected with the oeZNF304, 183-5p inhibitor or co-transfected with both and then subjected to western blot analysis the FOXO4 and Cyclin D1 protein level. **(C, D)** Indicated RNA and vectors were used to transfect into 786-0 cells and the FOXO4 and Cyclin D1protein level were detected by Western blot. **(E–G)** xenograft tumors were performed by injecting 786-0 cells with stably downregulated miR-183-5p in nude mice and tumor volumes **(E, F)** or tumor weight **(G)** were measured by direct measurement. **(H)** FOXO4 and Cyclin D1 proteins level in xenograft tumors were examined by using western blot. All data are from three independent experiments and expressed as the mean ± SEM. *P < 0.05, **P < 0.01 vs. the corresponding controls.

Overexpression of ZNF304 increased FOXO4 protein level and reduced cyclin D1 level, and the regulation of FOXO4 was enhanced by miR-183-5p inhibitor co-transfection (**Figures 6A, B**). Depletion of FOXO4 promoted cyclin D1 expression, while miR-183-5p inhibitor co-transfection reversed the promotion of FOXO4 (**Figures 6C, D**). Finally, we used a xenograft model to examine whether disruption of ZNF304/miR-183-5p/FOXO4 axis inhibits cell growth *in vivo*. Injection of 786-0 cells with stable depletion of miR-183-5p, to block ZNF304/miR-183-5p/FOXO4 axis, yielded smaller tumors in nude mice than in those injected with sham-transfected cells (**Figures 6E, F**). Similarly, stable knockdown of miR-183-5p also resulted in a smaller wet tumor weight (**Figure 6G**). Western blot analysis also revealed that silence of miR-183-5p significantly increased FOXO4 but reduced cyclin D1 proteins level compared to tumors derived from control cells (**Figure 6H**). These findings suggest that disruption of ZNF304/miR-183-5p/FOXO4 axis inhibits cell proliferation in ccRCC cells *in vivo*.

## Discussion

**Figure 7:**
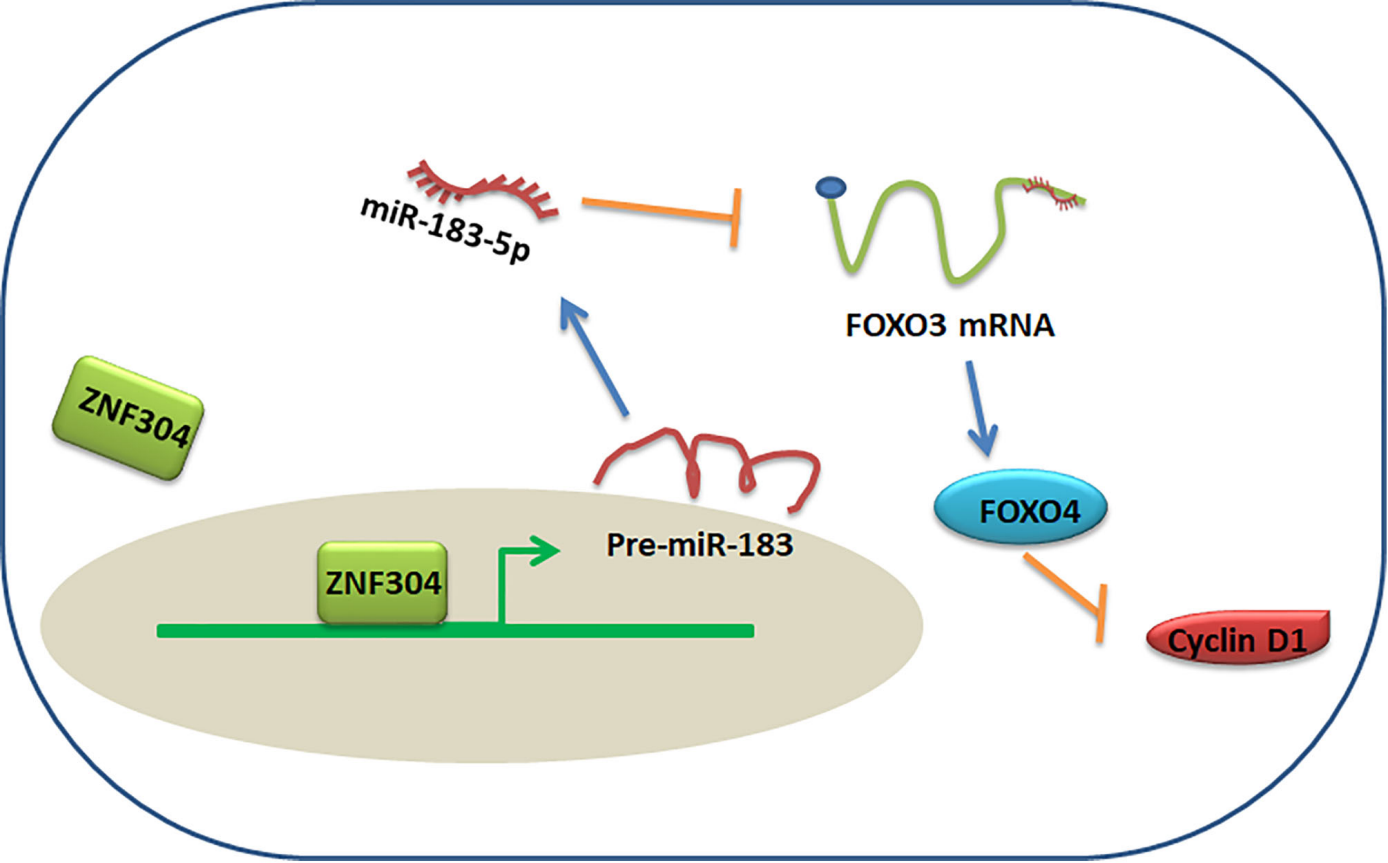
Proposed model for ZNF304/miR-183-5p/FOXO4 regulation of ccRCC progression.

The main discovery in this study is that ZNF304, which is significantly decreased in ccRCC tissues, acts as a critical upstream regulator of FOXO4. Overexpression of ZNF304 resulted in antitumor activity and inhibited RCC cell growth *in vitro* through FOXO4 upregulation. Low level of ZNF304 mRNA was associated with poor survival of ccRCC patients. Interestingly, ZNF304 positively regulated FOXO4 protein expression but not mRNA level. Mechanistically, ZNF304 bound to miR-183 promoter and activated miR-183-5p transcription. Upregulation of miR-183-5p directly bound to 3′UTR of FOXO4 and repressed its expression (**Figure 7**). Our findings suggest ZNF304/miR-183-5p/FOXO4 pathway plays a vital role in regulation of cell survival in ccRCC.

The occurrence and development of tumors is a complex process involving multi-step, multi-stage, and multi-factor regulation (31). This process also involves the abnormal expression of many genes, such as the upregulation of proto-oncogenes and the downregulation of tumor suppressor genes (12–15). However, many current studies have focused on upregulated genes in tumors, and have ignored the research on downregulated genes. In fact, these abnormal genes play an equally important role in the tumor process (16). On the technical level, it is easier for researchers to develop targeted inhibitors or targeted suppression of upregulated genes than targeted downregulated genes. However, genes whose expression is downregulated can also achieve tumor suppression through the development of agonists or targeted upregulation. In the present study, we demonstrated that ZNF304 downregulated in ccRCC and downregulation of ZNF304 promoted expression of miR-183-5p which is a upstream molecular of FOXO4. *In vivo* and *in vitro* results indicate that overexpression of ZNF304 or targeted inhibition of miR-183-5p can effectively inhibit tumor progression. Therefore, development of ZNF304 or FOXO4 agonists or the use of technologies such as CRISPR-Cas9 (32, 33) to target upregulation of these genes expression, or the use of miR-183-5p antagonists are expected to achieve the inhibition of tumor progression. Therefore, we believe that the downstream pathway of ZNF304 is possible as a potential anti-tumor pathway. We plan to explore the upstream regulator of ZNF304 and FOXO4 in our next study.

FOXO4, as a transcription inhibitor, one of its important functions is to inhibit tumor growth (21). Downregulation of FOXO4 in nasopharyngeal carcinoma is significant and related to distant metastasis, tumor recurrence and low overall patient survival rate (34). Concomitantly, the expression of FOXO4 is negatively correlated with the level of miR-150, and overexpression of FOXO4 significantly reduces tumor cell invasion *in vitro* (34). FOXO4 overexpression inhibits breast cancer cell growth and delays the occurrence and development of tumors in nude mice (35). Cholangiocarcinoma cell growth *in vitro* is repressed by FOXO4 *via* induction of G0/G1 arrest (36). FOXO4 also limits glioblastoma development by inhibiting the malignant phenotype of cells (25). In addition, FOXO4 has been shown to inhibit cervical cancer, gastric cancer, liver and nasopharyngeal cancer cell growth and tumor progression (37–40). However, little is known about the function of FOXO4 in ccRCC.

miRNA participates in a variety of biological processes through post-transcriptional regulation, including cell growth, migration and invasion, cell cycle progression and apoptosis (41). Abnormally expressed miRNAs may have an oncogene or tumor suppressor effect, often depending on the biological function of its target (42). In previous studies, we found that miR-193a-5p silencing inhibited the resistance of prostate cancer cells to docetaxel (29). miR-193a-5p mediates the p53-RBM25-circAMOTL1L axis to regulate Pcdha expression in the epithelial-mesenchymal transition of prostate cancer cells (30, 43). Our recent study found that miR-212-5p/miR-449a inhibits the expression of E2F5 and mediates circCDK13/CDK13 feedback regulation to promote the occurrence and development of prostate cancer (28). Depletion of miR-183-5p reduces radio-resistance in colorectal cancer through direct retinal ATG5 (44). In addition, miR-183-5p facilitates growth and metastasis of hepatocellular carcinoma cells by targeting IRS1 and is associated with patient survival (45). Similar to our study, Li et al. reported that higher level of miR-183-5p was associated with poor survival of patients after RCC surgery (46). In the present study, we demonstrated that level of miR-183-5p was significantly elevated in ccRCC tissues compared to normal kidney tissues. The higher level of miR-183-5p predicted poor overall survival of patients with ccRCC. By targeting 3′UTR of FOXO4, miR-183-5p inhibited FOXO4 expression in RCC cells. Interestingly, ZNF304, which is a upstream regulator of FOXO4, bound to miR-183 promoter and repressed miR-183-5p transcription.

## Conclusion

In summary, our results demonstrated that downregulation of ZNF304 affected miR-183-5p/FOXO4 axis, further repressing cell growth in ccRCC. These data provide theoretical evidence that ZNF304/miR-183-5p/FOXO4 pathway plays a critical role in ccRCC progression. Therefore, a comprehensive understanding of the mechanism responsible for regulating this axis will promote the development of an effective therapeutic strategy to inhibit ccRCC progression.

## Data Availability Statement

The original contributions presented in the study are included in the article/**Supplementary Material**. Further inquiries can be directed to the corresponding authors.

## Ethics Statement

The studies involving human participants were reviewed and approved by The Ethics Committee of the Second Hospital of Hebei Medical University. The patients/participants provided their written informed consent to participate in this study.

## Author Contributions

ZY, J-FG, and L-XR contributed to conception and design of the study. L-XR, BS, HZ, D-DW, and MZ organized the database. B-WZ performed the statistical analysis. L-XR and ZY wrote the first draft of the manuscript. ZY, J-FG, B-WZ, and L-XR wrote sections of the manuscript. All authors contributed to the article and approved the submitted version.

## Funding

This study was partially supported by The National Natural Science Foundation of China (No. 81970216, 82072842), Excellent Youth Science Foundation of Hebei Province (H2019206536).

## Conflict of Interest

The authors declare that the research was conducted in the absence of any commercial or financial relationships that could be construed as a potential conflict of interest.

## Publisher’s Note

All claims expressed in this article are solely those of the authors and do not necessarily represent those of their affiliated organizations, or those of the publisher, the editors and the reviewers. Any product that may be evaluated in this article, or claim that may be made by its manufacturer, is not guaranteed or endorsed by the publisher.

## Abbreviations

ZNF304, Zinc-finger protein 304; ccRCC, clear cell renal carcinoma; ChIP, Chromatin immunoprecipitation; RCC, Renal cell carcinoma; FOXO4, Forkhead box O 4; CRC, colorectal cancer; mRCC, Metastatic renal cell carcinoma; miR, microRNA; RT-qPCR, Real-time quantitative PCR; WT, wild-type; MTT, 3-(4,5-dimethylthiazol-2-yl)-2,5-diphenyltetrazolium bromide; shZNF304, short hairpin RNA ZNF304; shFOXO4, short hairpin RNA FOXO4; oeZNF304, overexpression ZNF304.
