# Supplementary material for: A Novel ZNF304/miR-183-5p/FOXO4 Pathway Regulates Cell Proliferation in Clear Cell Renal Carcinoma
Source: Front Oncol. 2021 Oct 7;11:710525. doi: 10.3389/fonc.2021.710525 (PMC8529286; doi:10.3389/fonc.2021.710525)
Supplement: Supplementary file 1 [file Image_1.pdf]

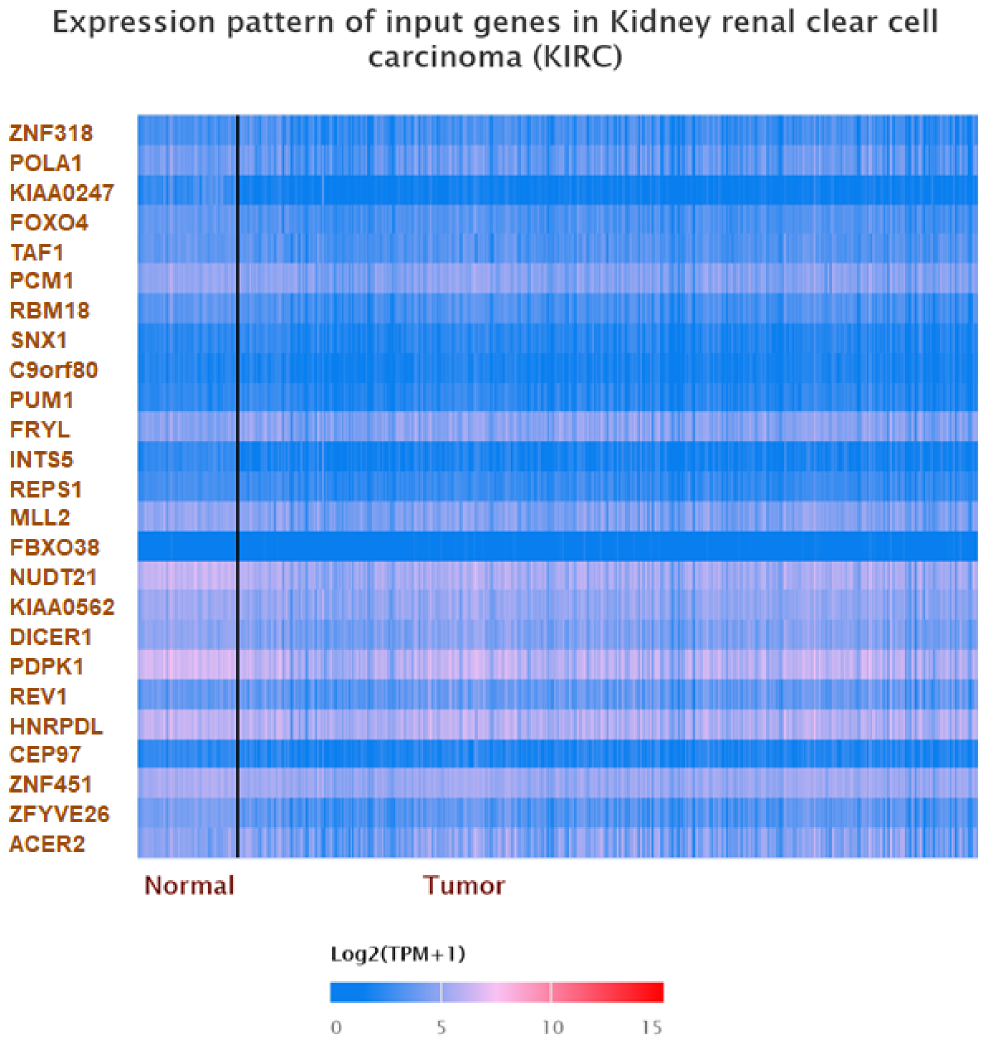

Supplementary Figure 1. Analyzed some genes positively related to ZNF304 expression in ccRCC tissues from the TCGA database.
