# Supplementary material for: A Novel ZNF304/miR-183-5p/FOXO4 Pathway Regulates Cell Proliferation in Clear Cell Renal Carcinoma
Source: Front Oncol. 2021 Oct 7;11:710525. doi: 10.3389/fonc.2021.710525 (PMC8529286; doi:10.3389/fonc.2021.710525)
Supplement: Supplementary file 2 [file Image_2.pdf]

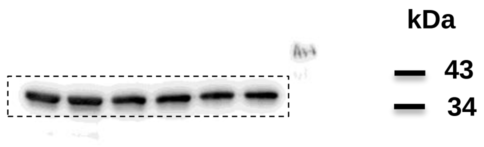

β-actin

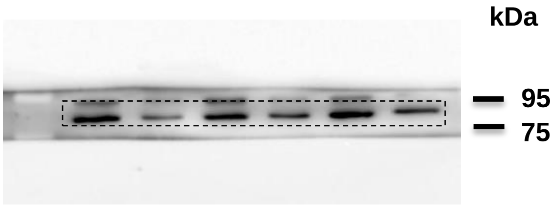

ZNF304

Supplementary Figure 2. The original immunoblots of Figure 1C.

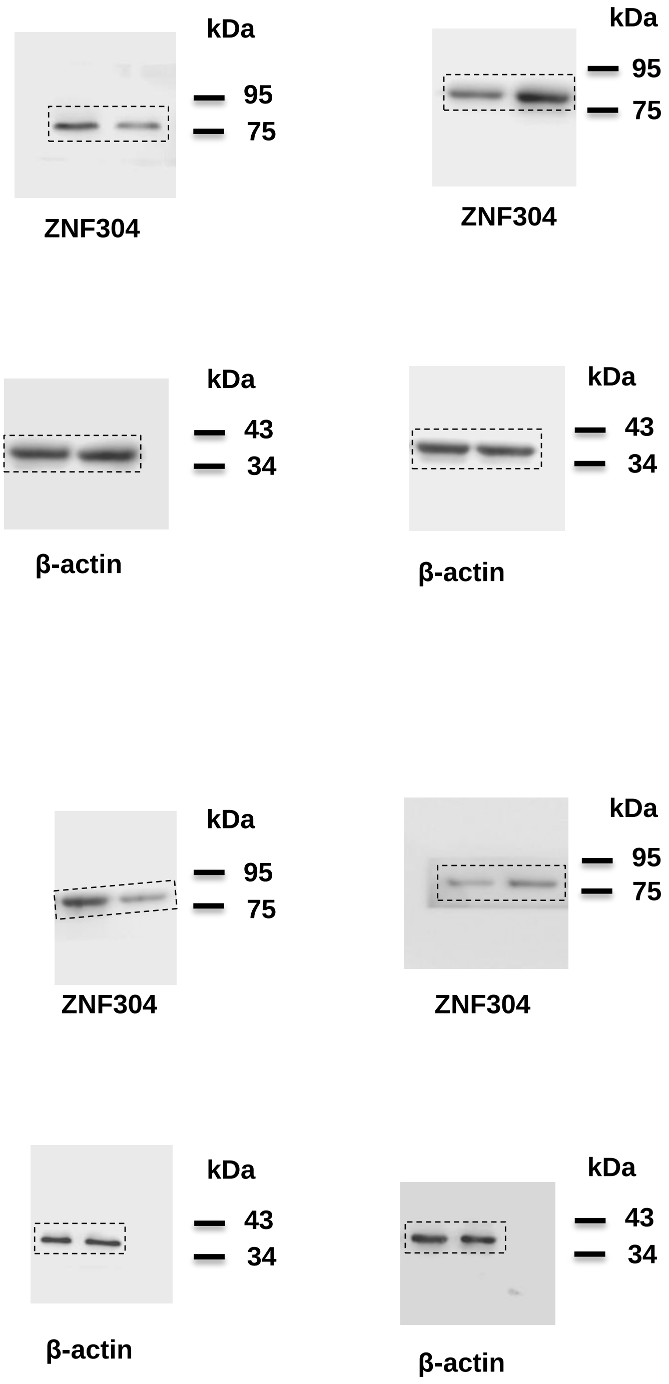

Supplementary Figure 3. The original immunoblots of Figure 2B.

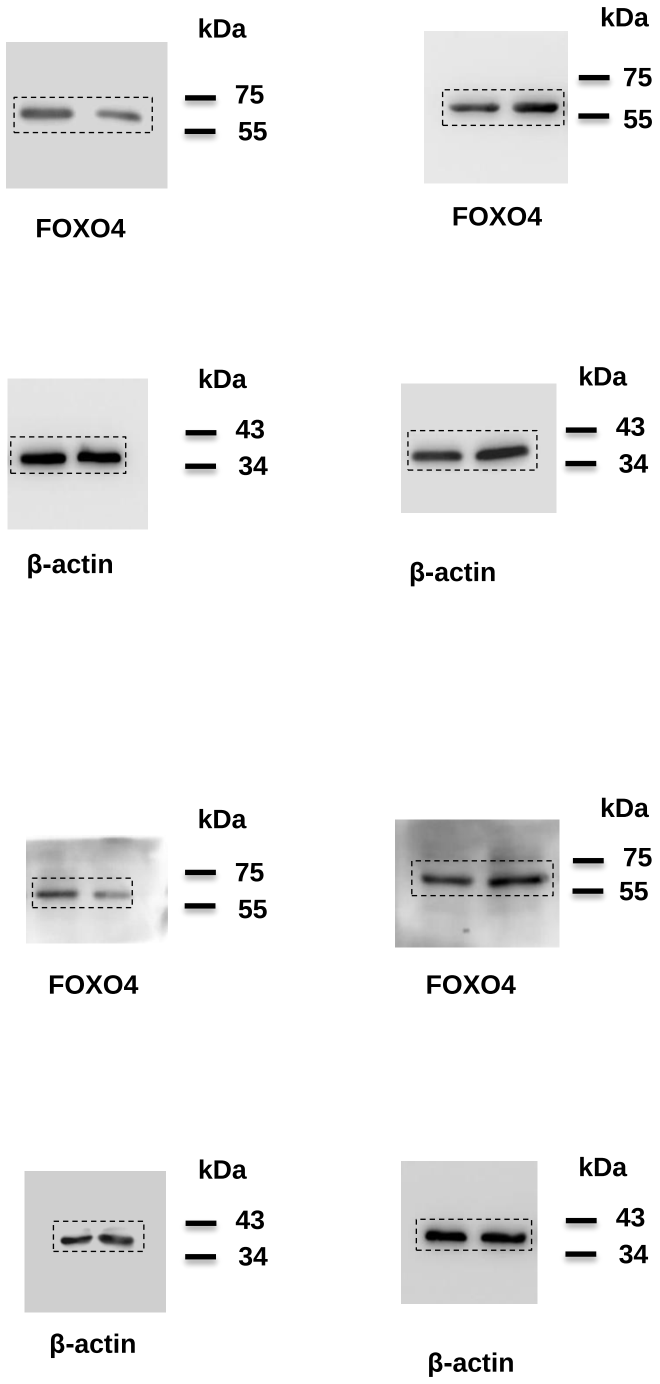

Supplementary Figure 4. The original immunoblots of Figure 3A.

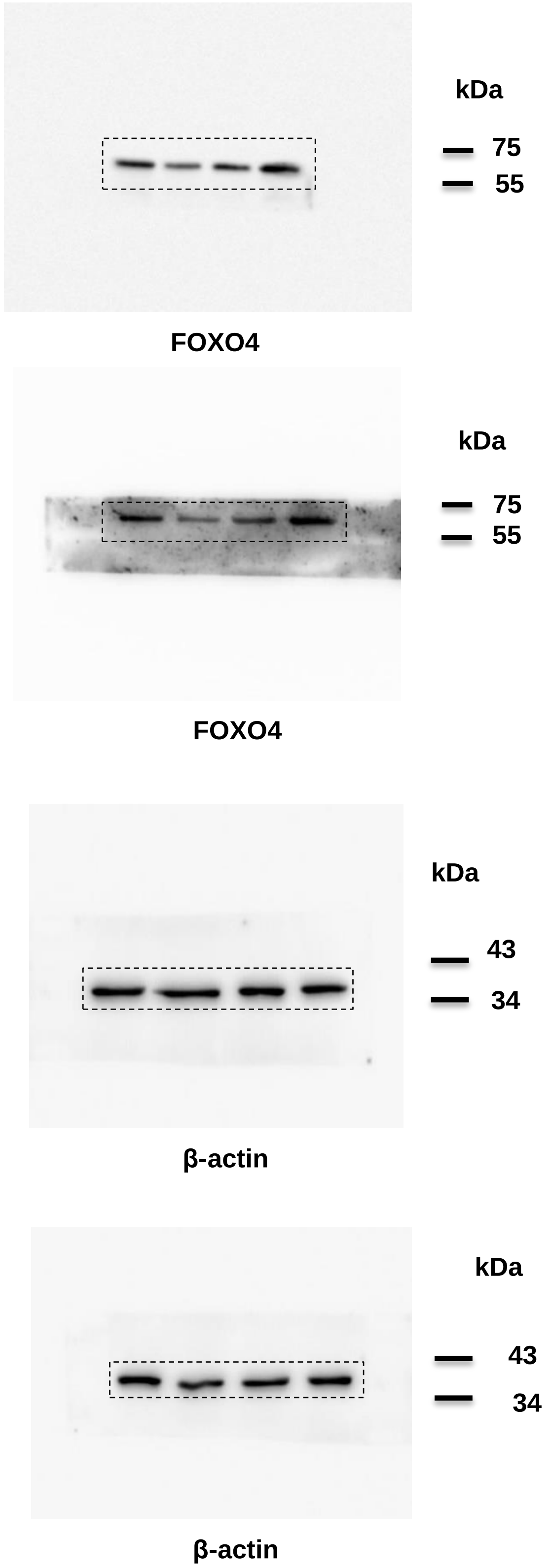

Supplementary Figure 5. The original immunoblots of Figure 4F.

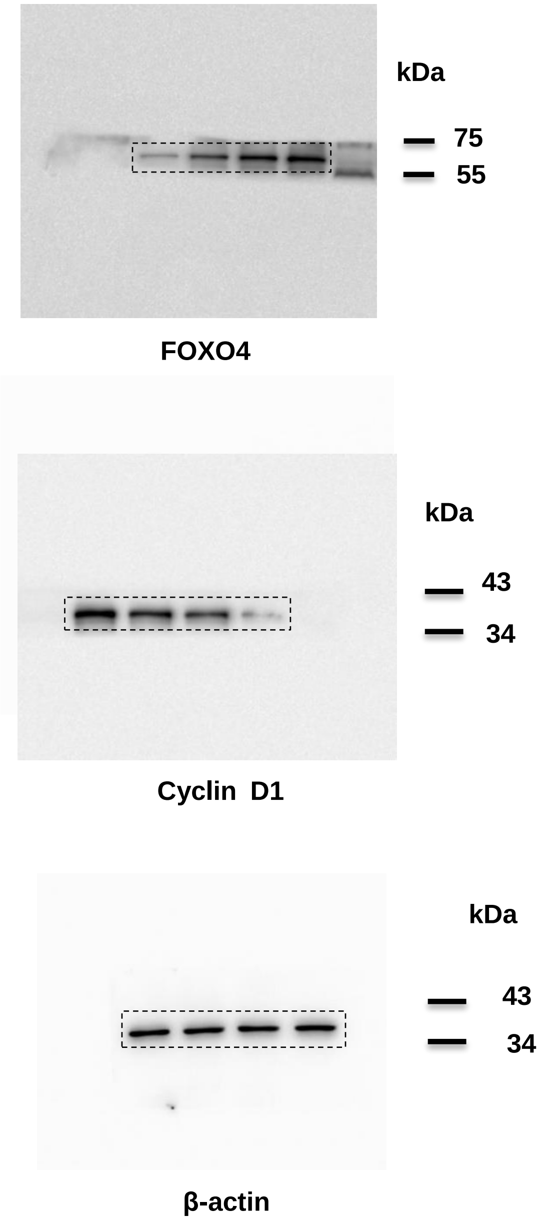

Supplementary Figure 6. The original immunoblots of Figure 6A.

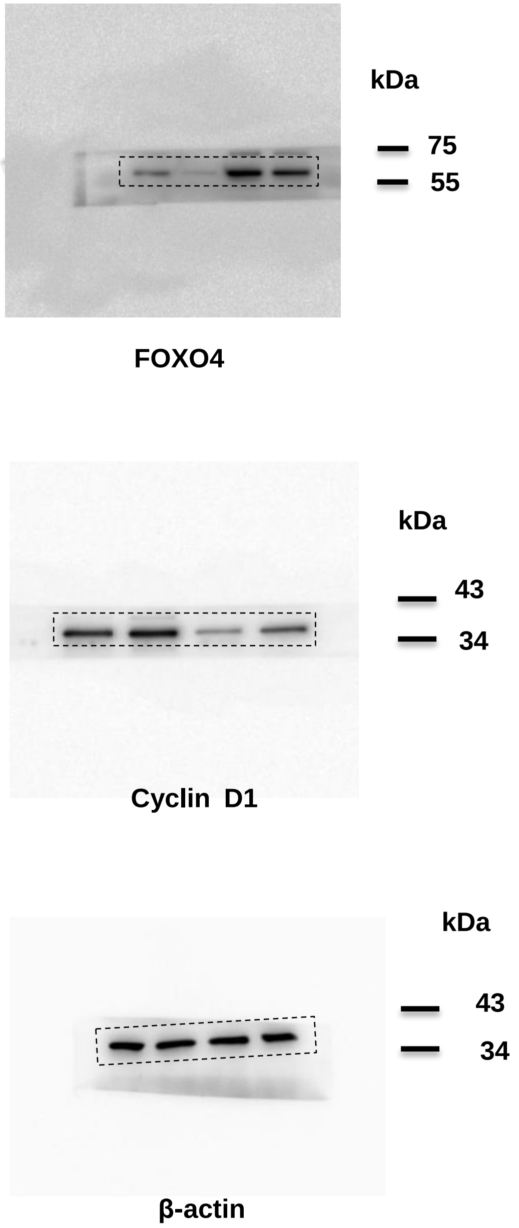

Supplementary Figure 7. The original immunoblots of Figure 6C.

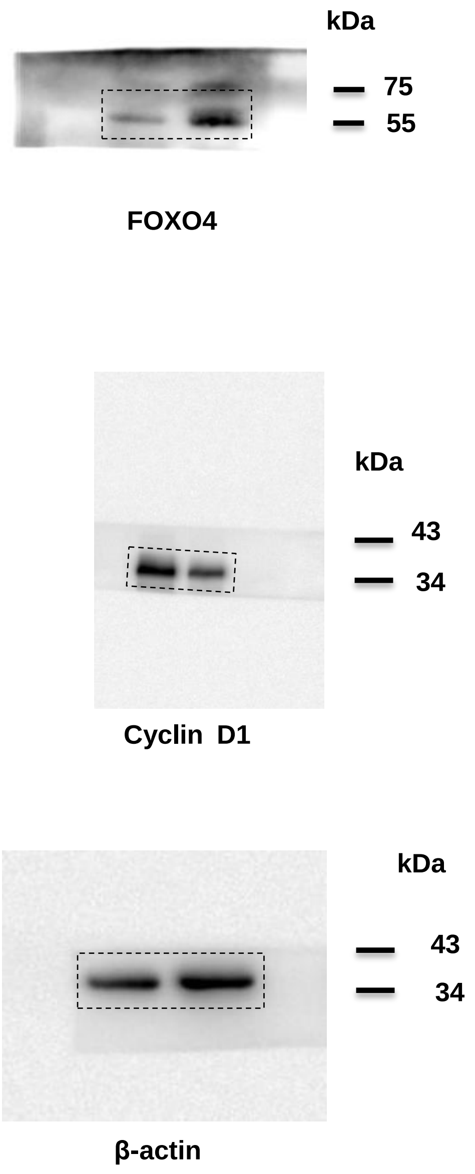

Supplementary Figure 8. The original immunoblots of Figure 6H.
